# Recent Advances in the Discovery of CK2 Allosteric Inhibitors: From Traditional Screening to Structure-Based Design

**DOI:** 10.3390/molecules25040870

**Published:** 2020-02-16

**Authors:** Xiaolan Chen, Chunqiong Li, Dada Wang, Yu Chen, Na Zhang

**Affiliations:** 1 Jiangsu Agri-animal Husbandry Vocational College, Taizhou 225300, China; wangdada860926@163.com (D.W.); medicalchenyu@126.com (Y.C.); 2 Beijing Key Laboratory of Environmental & Viral Oncology, College of Life Science and Bioengineering, Beijing University of Technology, Beijing 100124, China; chunqiong.li@emails.bjut.edu.cn

**Keywords:** protein kinase (CK2), allosteric site, allosteric inhibitors, traditional screening

## Abstract

Protein kinase (CK2) has emerged as an attractive cancer therapeutic target and recent efforts have been made to develop its inhibitors. However, the development of selective inhibitors remains challenging because of the highly conserved ATP-binding pocket (orthosteric site) of kinase family. As an alternative strategy, allosteric inhibitors, by targeting the much more diversified allosteric site relative to the conserved ATP-binding site, achieve better pharmacological advantages than orthosteric inhibitors. Traditional serendipitous screening and structure-based design are robust tools for the discovery of CK2 allosteric inhibitors. In this review, we summarize the recent advances in the identification of CK2 allosteric inhibitors. Firstly, we briefly present the CK2 allosteric sites. Then, the allosteric inhibitors targeting the well-elucidated allosteric sites (α/β interface, αD pocket and interface between the Glycine-rich loop and αC-helix) are highlighted in the discovery process and possible binding modes with the allosteric sites are described. This study is expected to provide valuable clues for the design of CK2 allosteric inhibitors.

## 1. Introduction

Protein kinase (CK2) is a ubiquitous Ser/Thr kinase that phosphorylates more than 300 substrates and is also involved in several cellular processes as the cell survival promoter and apoptosis suppressor [1,2]. The CK2 holoenzyme is often described as a tetramer of two catalytic subunits (CK2α or CK2α’) and two regulatory subunits (CK2β).Interestingly, the CK2 holoenzyme complex is subject to disassembly and reassembly, andthe irreversible nature of the CK2 holoenzyme makes the free population of each subunit, CK2α(orCK2α’) and CK2β,co-exist in different cellularcompartments [3]. Unlike other multi-subunit protein kinases, CK2β is not required for the activity ofthe catalytic subunits. Both the free catalytic subunit CK2α (or CK2α’) and the CK2 holoenzyme complex are endowed with constitutive activity, while CK2β acts only as a targeting subunit and/or the central component of the stable tetrameric CK2 complex, affecting full enzymatic activity, stability, and CK2β-dependent substrate specificity. Hence, a limited number of protein substrates (eIF2β) are phosphorylated by the CK2 holoenzyme complex, but not the noncomplexed CK2α [4]. Meanwhile, CK2 is also involved in the regulation Wnt signaling, NF-κB and PTEN/PI3K/Akt-PKB pathways by stabilization of the pro-oncogene and anti-apoptosis factors. Mounting evidencehasdemonstrated that over-expressed CK2 iscorrelatedwith many commonsolid tumor types, which include lung cancer, head and neck cancer, bladder cancer, or mesotheliom [5]. Consequently, this kinase has been regarded as avaluable therapeutic target, and numerous efforts have been made towardsthe discovery of CK2 inhibitors for cancer therapy.

The growing amount of CK2 crystal structures inthe Protein Data Bank provide valuable clues for the discovery of CK2 inhibitors. Diverse strategies to inhibit CK2 functions by small moleculeshave been explored in preclinical studies. By targeting the ATP-binding pocket, substrate-binding site, allosteric sites or CK2α/β interface, a variety of CK2 inhibitors with the anti-cancer activity have been developed [6]. Like other kinases, the ATP-binding pocket of CK2α has been considered as the orthosteric site to design ATP-competitive inhibitors, such as benzimidazole-derivative anthraquinone, tricyclic quinolone derivatives and natural products [7,8,9]. Unfortunately, although several ATP-competitive inhibitors of CK2, ranging from micromolar to nanomolar, have been discovered, most of them were impeded in their ability to become the promising drug candidates due to their lower specificity and diversity deficiencies [10]. Despite the fact thatthe potent compound CX-4945 (IC_50_ = 1.5 nM against CK2α) has entered phase II clinical trials for the treatment of cholangiocarcinoma [11], this compound still suffered from low selectivity (dual-specificity tyrosine phosphorylation-regulated kinases (Dyrk) 1A and 1B with IC_50_ values of 6.8 and 6.4 nM, respectively) due to the highly conserved ATP-binding pocket of kinases [12]. Therefore, to overcome the general shortcomings, an alternative strategy strives to identify non-ATP-competitive inhibitors targeting sites outside of the conserved ATP site.

**Figure 1:**
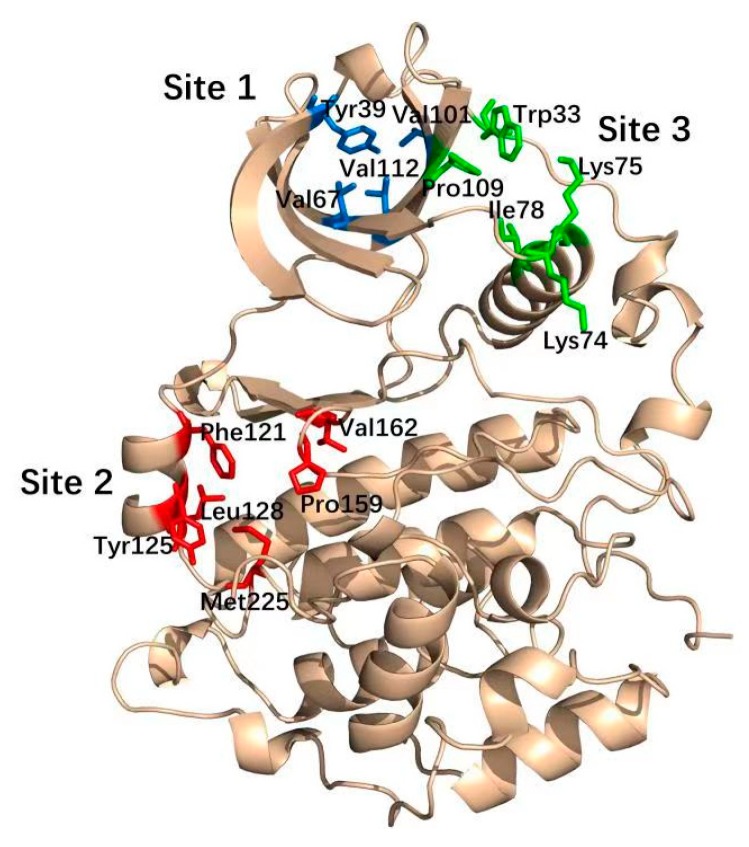
Three well-elucidated allosteric sites of CK2α including Site 1 (blue), Site 2 (red) and Site 3 (green).

Allostery is the regulation of macromolecule function through the binding of a modulator to an allosteric site thatis spatially and topographically distinct from its orthosteric site. Therefore, allosteric sites areregarded as attractive targets to develop non-ATP-competitive kinase inhibitors [13,14,15]. By targeting the much more diversified allosteric site relative to the highly conserved ATP-binding pocket, allosteric inhibitors might avoid the drawbacks of most conventional ATP-competitive inhibitors with theirenhanced selectivity and reduced toxicity. For example, the subtype selective PDE4D allosteric inhibitor BPN14770 exhibited reduced vascular toxicity over earlier PDE4 inhibitors that lacked the subtype selectivity [16], and compound 8t, as the allosteric inhibitor of phosphoglyceratemutase 1, was demonstrated to delay tumor growth in the H1299 xenograft model without the obvious toxicity [17]. Notably, the identification of CK2 allosteric sites is a prerequisite tothe discovery of allosteric inhibitors. To date, six allosteric sites of CK2α have been identified using the combination of bioinformatics and biochemistry methods [18], and three well-elucidated sites among them have been targeted to discover CK2 allosteric inhibitors. As shown in Figure 1, Site 1, α/β interface (Tyr 39, Val67, Val112 and Val101) has been confirmed to be occupied by the DRB, cyclic peptide (Pc) and W16 [19]. The αD pocket named Site 2 was demonstrated as the allosteric pocket including Phe121, Pro125, Leu128, Pro159, Val162 and Met225. Recently, Bestgen et al. discovered aminothiazole derivatives as the allosteric modulators of CK2 by targeting the interface between the αC helix and the glycine-rich loop (G-loop) [20].

Most of the known CK2 allosteric hits were discovered by high-throughput screening (HTS) and virtual screening methods, which are the useful approaches to identify starting points against any target of interest for drug design [21,22]. This review highlights the recent progress in the development of CK2 allosteric inhibitors. We classified these inhibitors according to the allosteric sites where they bind, including Sites 1,2 and 3, and presented their discovery method and the key receptor–ligand interactions at the allosteric sites in detail.

## 2. Compounds Targeting Site 1

**Figure 2:**
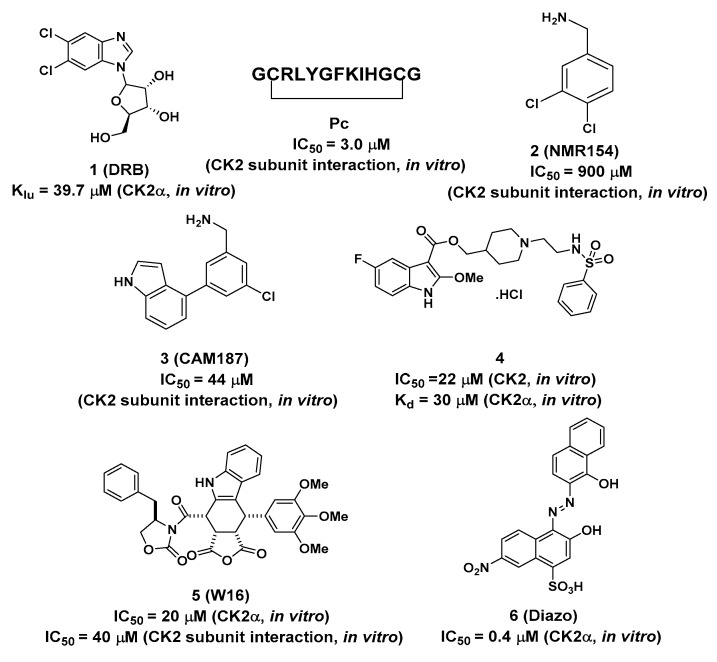
Structures of compounds (**1**, Pc, **2**, **3** and **4**) and allosteric inhibitors (**5–6**) binding to Site 1.

As indicated from the crystal structures of CK2 holoenzyme, (PDB ID: 1JWH) [23] and (PDB ID: 4IB5) [24], the subunit interface is located at a shallow groove consisting of hydrophobic residues of the G-, C-, and β4/β5 loops of CK2α *N*-terminal domain. These observations provide a strategy to identify inhibitors that modulate interactions between the CK2α/CK2β subunits as indicated in Figure 2. Although this interface was predicted as the druggable allosteric site, not all the molecules that bind here are the allosteric inhibitors. 

DRB was found to bethe first small molecule with adual binding mode, by targeting the ATP pocket and α/β interface simultaneously (PDB ID: 2RKP) [25]. This discovery presented a new druggable site out of the catalytic pocket on CK2α, with the potential toinducethe selective disruption of the CK2α/CK2β subunit assembly. However, kinetic measurements indicated that the second DRB only disturbed the formation of CK2α/CK2β holoenzyme, without any inhibitory effects on CK2α.

**Figure 3:**
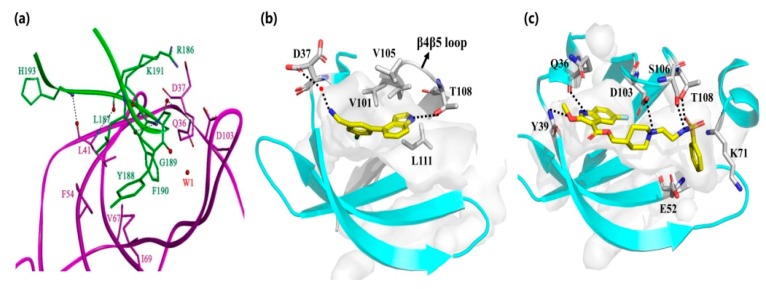
Interaction between (**a**) Pc, (**b**) compound **3** and (**c**) **4** with α/β interface.

Laudet et al. [26] designed a truncated CK2β mimic peptide (Pc) with the ability to antagonize the assembly of the CK2 holoenzyme through competition with CK2β binding to CK2α. As shown in Figure 3, the hydrophobic interactions between the hotspot amino acids Y188 and residues F54 of the G-loop and I69 of the C-loop, as well as F190 with residues Q36 and L41 of the G-loop, V67 of the C-loop, and Val101 of the β4/β5 loopall contributed to the binding of Pc to CK2α. Our group also revealed the dynamic structural features of CK2α-Pc complex with the single mutation of Y188A and F190A [27]. By analyzing the contribution of key resides indicated from molecular dynamics simulations, a series of peptides with animproved binding affinity to CK2α wererationally designed and synthesized, as well as anti-proliferative and pro-apoptotic effects against the HepG2 cell line [28]. Additionally, the introduction of halogens into the meta-position of F190 [29] or a novel covalent linker on Pc [30] have been explored as the possibleimproved versions. Unfortunately, none of the Pc derivatives are cell-permeable, and, thus, they all require a cell-penetrating peptide for delivery.

Fragment-based screening is also another powerful tool for the development of allosteric inhibitors. By screening the fragment library, an initial hit compound, 2 (NMR154), was found to bind in both the ATP pocket and the α/β interface of CK2 with an IC_50_ of 900 μM [31]. Through elaborate iterations, compound **3** (CAM187) was synthesized and exhibited potent inhibitory activity on CK2 (IC_50_ = 44 μM) with the improving selectivity for α/β interface binding only. As indicted from the co-crystal structure of CK2α-CAM187, the biaryl part of this molecule fitted into the α/β interface. The amine formed apolar interaction with D37 and the indole ring located under the β4/β5 loop, alongside the interaction between the nitrogen atom and the OH group of T108. Due to the better drug-like properties of CAM187 relative to W16 and Diazo, the potent and selective fragment CAM187 could be regarded as apharmacophoric group for the development of drug-like molecules.

In parallel with efforts to develop peptide-based inhibitors, compound **4** was optimized and validated to bind at the α/β interface based on the lead compound discovered by virtual ligand docking and screening [32]. As shown in Figure 3, the fluorinated indole ring was buried in the hydrophobic pocket consisting of L41, F54, V67, V101, P104, V105, A110, and V112. Additionally, Q36 and Y39 formed polar interactions with the -NH- of the indole ring and the oxygen atom of ester, respectively. The left part, linked with ester, extended toward the top of the kinase P-loop with a salt bridge interaction between the nitrogen of the piperidine ring andthe carbonyl of D103, as well as the hydrogen bond between sulfonamide and S106 and T108 of the β4–β5 loop (Figure 3b). Enzymatic assay confirmed that compound 4 blocked the CK2α/CK2β interaction and inhibited phosphorylation of CK2β-dependent substrates. Furthermore, this compound exhibited better cell permeability than Pc with the impeded cell growth, migration and induced cell death effects on triple-negative breast cancer cells (MBA-MB-231). Therefore, this compound is the first example of a rationally designed small molecule that efficiently displaces CK2β from CK2α in the cellular context.

DRB, Pc and compounds **3** and **4** only efficiently inhibited the CK2-holoenzyme mediated phosphorylation of CK2β-dependent substrates, but without inhibitory effects on the catalytic activity of CK2α. Consequently, these mentioned compounds may not be considered the real allosteric inhibitors, but the allosteric site-binding molecule.

By a high-throughput screening of a collection of phyllotoxineindolo-analogues, W16wasidentified as the first chemical inhibitor that showedselective disruption of the CK2α/CK2β assembly through binding to the CK2α/CK2β interface [33]. Inhibition kinetic studies revealed that W16 induced a non-ATP-competitive inhibition of CK2α. Unlike Pc, the binding of W16 into the subunit interface may trigger a functional conformational change in CK2α that exerted a negative influence on the ATP-binding site.

Azonaphthalene derivatives were also discovered as newly non-ATP-competitive CK2 inhibitors using an automated screening method, and compound **6** (Diazo) displayed an IC_50_ of ~0.4 μM on CK2α [34]. Small-angle X-ray scattering experiments showed that this inhibition was due to the inactive conformation of CK2α that blocked the binding of substrates upon the binding of these inhibitors. Diazo (at 5 μM) selectively inhibited more than 95% ofCK2 but had no effect on the other protein kinases tested, such as Pim-1, CDK2 and JAK2.However, as it was not possible to solve the crystal structure of CK2α-Diazo, further structural analyses are still needed to reveal the inhibition mechanisms.

Overall, although all the mentioned compounds interfered with the specific interaction between CK2α and CK2β, the inhibitory effects of them exerted on CK2 were different. Contrary to ATP-competitive inhibitors, DRB, Pc and compound **3** and **4** only inhibited the phosphorylation of CK2β-dependent substrates without affecting the catalytic activity of CK2α. However, the binding of W16 and Diazointo the CK2α/β interface may have triggereda negative effecton the active conformation of CK2αthat blocked the binding of ATP or substrates and, thus, inhibited the catalytic activity of CK2α.

## 3. CAM4066 Derivatives and Uracil AnaloguesTargeting Site 2

**Figure 4:**
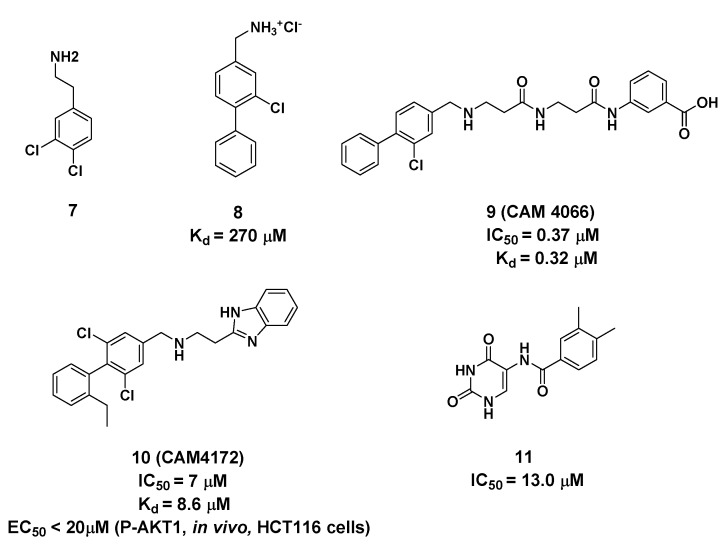
Structure and biological activity of compounds targeting Site 2 (K_d_ and IC_50_ values tested in vitro).

**Figure 5:**
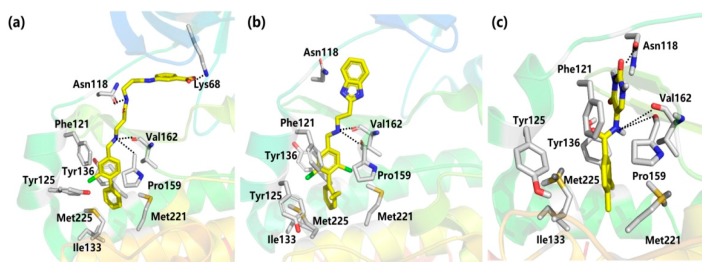
Interaction modes of compounds (**a**) **9** (CAM4066), (**b**) **10** (CAM4712) and (**c**) **11** with αD pocket.

Using a high concentration crystallographic screen method, compound **7** was identified asbinding in the αD site, but with lower Kd values (Figure 4) [35]. The amine formed hydrogen bonds with the backbone carbonyls of V162 and P159, and the phenyl were accommodated into the hydrophobic pocket. Based on compound **7**, the replacement of the chlorine atom at Position 4 with a phenyl ring was optimized to make it bind more deeply into the αD pocket. As a result, compound **8** showed the higher affinity of 270 μM, although without any inhibition of CK2 kinase activity. With the introduction of the optimized flexible linker combining two pharmacophoric fragments, CAM4066 was synthesized and showed potent inhibitory effects and good selectivity on CK2α (IC_50_ = 0.37 μM) [36]. As shown in Figure 5, the two pharmacophoric fragments were involved in forming hydrogen bonds with Pro159, Val162 and Lys68, respectively, while the linker formed the H-bonds with Asn118. Appropriate coupling between the linker and pharmacophore fragments was essential for the binding of CAM4066 with CK2α. Our group explored the structural mechanism for the decreasedinhibitionof the rigid linker and non-ionizable substituted fragment (pre-CAM4066) against CK2 by molecular dynamics simulation (MD) studies [37].

However, becauseCAM4066 interacted with the αD pocket (Figure 5) and the positive area of the ATP-binding pocket simultaneously, despite its potent inhibition effect and high selectivity, this compound may not be considered as aclassic allosteric inhibitor which binds completely outside the orthosteric pocket. With the aimofdeveloping improved αD-site binding inhibitors without reaching deep into the ATP-binding pocket, the second generation inhibitor CAM4712 (IC_50_ = 7 μM) [38] was designed by growing benzimidazole fragments near, but not inside, the mouth of ATP sites, which is are markable improvement relative to CAM4066. Similar to compound CAM4066, NH was entrapped in the hydrophobic site and formed an H-bond with Pro159 and Val162. Meanwhile, the benzimidazole fragment formed the π–π interaction with His160. Competition experiments further confirmed that CAM4712 was the potent and selective allosteric inhibitor by binding in the αD pocket without facilitatingcellular uptake.

Besides the above mentioned αD-site binding inhibitors CAM4066 and CAM4712, our group identified the novel allosteric inhibitors of CK2α using the pharmacophore model and Alloscore-based virtual screening of ChemBridge fragment library [39]. By considering the filtration of fit values > 2.5 with the αD pocket pharmacophore model, as well as the Alloscore value of 5.8, compound **11** combined with the uracil group was discovered to be an allosteric inhibitor of CK2α (IC_50_ = 13.0 μM), which is similar to that of CAM4712. As indicated by the MD simulation result, the skeleton of this compound fitted well into the αD pocket, including the polar interaction formed between theuracil group and the NH linker withVal162 and Asn118, as well as the hydrophobic packing of a phenyl ring with residues Ile133, Tyr136, Met221 and Met225. Giventhe potent inhibitory effect of compound **11** against CK2α, we made afurther elaborate optimization ofthe linker and uracil substitution to discover the potent and selective CK2 allosteric inhibitors (unpublished manuscript).

Despite the fact that CAM4712 could bind in the αD pocket, and partially in the lip of the ATP site, this compound only showed good selectivity against the 20 closely related CMGC kinases in the panel at 30 μM, with the exception of four kinases (CAMK1, SmMLCK, EF2K and SGK1). Giventhat Site 2 is near the mouth of the ATP-binding pocket, further optimization of allosteric inhibitors only targeting the unique αD pocket is needed to improve selectivity towards CK2α.

## 4. 2-Aminothiazole Derivatives Targeting Site 3

**Figure 6:**
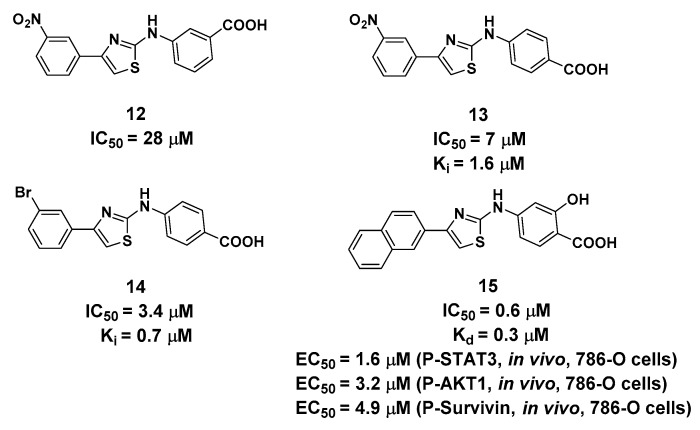
Structure and biological activity of 2-aminothiazole derivatives (Ki, Kd and IC_50_ values tested in vitro).

The serendipitous discovery of 2-aminothiazole derivatives (Figure 6) targeting the interface between the G-loop and αC-helixis very intriguing [20,40]. Originally, Lomberget et al. aimed to identify hits targeting the CK2α/CK2β interface using virtual ligand screening, and compound **12** was evaluated as the most active hit with an IC_50_ value of 28 μM for CK2α. However, elaborate biochemical experiments validated that compound **12** was not bound to the CK2α/CK2β interface, and its inhibitory effects on CK2 wereindependent of the concentrations of both ATP, peptide substrate, and the presence of CK2β. Further experiments were performed on compound **13**, which shared the same inhibition pattern as compound **12**, but with improved inhibitory effects (IC_50_ = 7.0 μM) and higher selectivity with CK2α.

**Figure 7:**
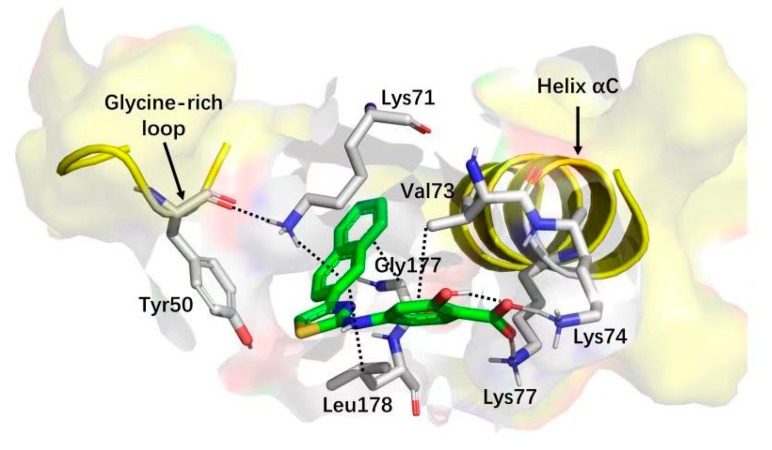
Cartoon/surface representation of the interaction between **15** with the G-loop and αC-helix.

The structure–activity relationship indicated that the aminothiazole core and a carboxylic acid function were essential for inhibitory activity. By combining the saturation transfer difference NMR competition experiments, alanine scan mutational analysis, the native mass spectrometry-based detection, and molecular docking, the 2-aminothiazole derivatives were predicted to bind in the interface between the G-loop and αC-helix by forming anH-bond, salt bridge (carboxylate with Lys74 and Lys77) or cation-π interactions (naphthalene ring with Lys71) as shown in Figure 7. After the systematical optimization on the position 4 and amine of the aminothiazole core, compound **15** was identified as having submicromolar potency against CK2α (IC_50_ = 0.6 μM) with pro-apoptotic and anti-cell growth effects. As Site 3 is less conserved in the human kinome, the overall selectivity of **15**, as expressed by a Gini coefficient of 0.78,was higher than that of CX-4945 (Gini coefficient of 0.67), in particular, the main off-targets of CX-4945, Clk2 and Dyrk1A were not or were only weakly inhibited by **15**, respectively.

## 5. Conclusions

The currently available ATP-competitive CK2 inhibitors were impeded from being the drug candidates due to their off-targets and low selectivity. The structural diversity of the allosteric sites endowed the allosteric inhibitors with improved pharmacological properties. This study outlined the recent process of allosteric inhibitors targeting three well-elucidated allosteric sites, including the α/β interface, αD pocket and the interface between the G-loop and αC-helix. Most of the mentioned allosteric inhibitors shared the common planar aromatic scaffold with the non-rotatable bonds, which was complementary to the narrow hydrophobic allosteric sites. However, as most of the CK2 allosteric hits were discovered by high-throughput and virtual screening methods, it is still necessary to engage in further exploration ofrational structural analysis and optimization, especially targeting the αD pocket and the interface between the G-loop and αC-helix.

## References

[1] Cozza G., Pinna L.A. (2016). Casein kinases as potential therapeutic targets. Expert Opin. Ther. Targets.

[2] Otto T., Sicinski P. (2017). Cell cycle proteins as promising targets in cancer therapy. Nat. Rev. Cancer.

[3] Olsen B.B., Guerra B., Niefind K., Issinger O.G. (2010). Structural basis of the constitutive activity of protein kinase CK2. Methods Enzymol..

[4] Filhol O., Giacosa S., Wallez Y., Cochet C. (2015). Protein kinase CK2 in breast cancer: The CK2b regulatory subunit takes center stage in epithelial plasticity. Cell. Mol. Life Sci..

[5] Chua M.M., Ortega C.E., Sheikh A., Lee M., Abdul-Rassoul H., Hartshorn K.L., Dominguez I. (2017). CK2 in cancer: Cellular and biochemical mechanisms and potential therapeutic target. Pharmaceuticals (Basel).

[6] Prudent R., Cochet C. (2009). New protein kinase CK2 inhibitors: Jumping out of the catalytic box. Chem. Biol..

[7] Cozza G. (2017). The development of CK2 inhibitors: From traditional pharmacoplogy to in silico rational drug design. Pharmaceuticals (Basel).

[8] Qiao Y., Chen T., Yang H., Chen Y., Lin H., Qu W., Feng F., Liu W., Guo Q., Liu Z. (2019). Small molecule modulators targeting protein kinase CK1 and CK2. Eur. J. Med. Chem..

[9] Oramas-Royo S., Haidar S., Amesty Á., Martín-Acosta P., Feresin G., Tapia A., Aichele D., Jose J., Estévez-Braun A. (2019). Design, synthesis and biological evaluation of new embelin derivatives as CK2 inhibitors. Bioorg. Chem..

[10] Cozza G., Meggio F., Moro S. (2011). The dark side of protein kinase CK2 inhibition. Curr. Med. Chem..

[11] Senhwa Biosciences, Inc. Senhwa Biosciences CX-4945 Granted Orphan Drug Designation by the US FDA in Cholangiocarcinoma. http://www.prnewswire.com/news-releases/senhwa-biosciences-cx-4945-granted-orphan-drug-designation-by-the-us-fda-in-cholangiocarcinoma-300385278.html.

[12] Kim H., Lee K.S., Kim A.K., Choi M., Choi K., Kang M., Chi S.W., Lee M.S., Lee J.S., Lee S.Y. (2016). A chemical with proven clinical safety rescues Down-syndrome- related phenotypes in through DYRK1A inhibition. Dis. Models Mech..

[13] Wenthur C.J., Gentry P.R., Mathews T.P., Lindsley C.W. (2014). Drugs for allosteric site son receptors. Annu. Rev. Pharmacol. Toxicol..

[14] Wu P., Clausen M.H., Nielsen T.E. (2015). Allosteric small-molecule kinase inhibitors. Pharmacol. Ther..

[15] Fang Z., Grütter C., Rauh D. (2013). Strategies for the selective regulation of kinases with allosteric modulators: Exploiting exclusive structural features. ACS Chem. Biol..

[16] Gurney M.E., Nugent R.A., Mo X., Sindac J.A., Hagen T.J., Fox D., O’Donnell J.M., Zhang C., Xu Y., Zhang H.-T. (2019). Design and synthesis of selective phosphodiesterase 4D (PDE4D) allosteric inhibitors for the treatment of fragile X syndrome and other brain disorders. J. Med. Chem..

[17] Huang K., Jiang L., Liang R., Li H., Ruan X., Shan C., Ye D., Zhou L. (2019). Synthesis and biological evaluation of anthraquinone derivatives as allosteric phosphoglycerate mutase 1 inhibitors for cancer treatment. Eur. J. Med. Chem..

[18] Jiang H.M., Dong J.K., Song K., Wang T.D., Huang W.K., Zhang J.M., Yang X.Y., Shen Y., Zhang J. (2017). A novel allosteric site in casein kinase 2α discovered using combining bioinformatics and biochemistry methods. Acta Pharmacol. Sin..

[19] Prudent R., Sautel C.F., Cochet C. (2010). Structure-based discovery of small molecules targeting different surfaces of protein-kinase CK2. Biochim. Biophys. Acta.

[20] Bestgen B., Krimm I., Kufareva I., Kamal A.A.M., Seetoh W.G., Abell C., Hartmann R.W., Abagyan R., Cochet C., Le Borgne M. (2019). 2-Aminothiazole derivatives as selective allosteric modulators of the protein kinase CK2. 1. identification of an allosteric binding site. J. Med. Chem..

[21] Wagner J.R., Lee C.T., Durrant J.D., Malmstrom R.D., Feher V.A., Amaro R.E. (2016). Emerging computational methods for the rational discovery of allosteric drugs. Chem. Rev..

[22] Lu S., He X., Ni D., Zhang J. (2019). Allosteric modulator discovery: From serendipity to structure-based design. J. Med. Chem..

[23] Niefind K., Guerra B., Ermakowa I., Issinger O.G. (2001). Crystal structure of human protein kinase CK2: Insights into basic properties of the CK2 holoenzyme. EMBO J..

[24] Raaf J., Guerra B., Neundorf I., Bopp B., Issinger O.G., Jose J., Pietsch M., Niefind K. (2013). First structure of protein kinase CK2 catalytic subunit with an effective CK2β-competitive ligand. ACS Chem. Biol..

[25] Raaf J., Brunstein E., Issinger O.G., Niefind K. (2008). The CK2a/CK2b interface of Human protein kinase CK2 harbors a binding pocket for small molecules. Chem. Biol..

[26] Laudet B., Barette C., Dulery V., Renaudet O., Dumy P., Metz A., Prudent R., Deshiere A., Dideberg O., Filhol O. (2007). Structure-based design of small peptide inhibitors of protein kinase CK2 subunit interaction. Biochem. J..

[27] Zhou Y., Zhang N., Chen W., Zhao L., Zhong R. (2016). Underlying mechanisms of cyclic peptide inhibitors interrupting the interaction of CK2α/CK2β: Comparative molecular dynamics simulation studies. Phys. Chem. Chem. Phys..

[28] Tang S., Zhang N., Zhou Y., Cortopassi W.A., Jacobson M.P., Zhao L.J., Zhong R.G. (2019). Structure-based discovery of novel CK2α-binding cyclic peptides with anti-cancer activity. Mol. Inform..

[29] Lindenblatt D., Horn M., Götz C., Niefind K., Neundorf I., Pietsch M. (2019). Design of CK2β-mimicking peptides as tools to study the CK2α/CK2β interaction in cancer cells. Chem. Med. Chem..

[30] Iegre J., Brear P., Baker D.J., Tan Y.S., Atkinson E.L., Sore H.F., O’Donovan D.H., Verma C.S., Hyvönen M., Spring D.R. (2019). Efficient development of stable and highly functionalised peptides targeting the CK2α/CK2β protein-protein interaction. Chem. Sci..

[31] Brear P., North A., Iegre J., Hadje Georgiou K., Lubin A., Carro L., Green W., Sore H.F., Hyvönen M., Spring D.R. (2018). Novel non-ATP competitive small molecules targeting the CK2 α/β interface. Bioorg. Med. Chem..

[32] Kufareva I., Bestgen B., Brear P., Prudent R., Laudet B., Moucadel V., Ettaoussi M., Sautel C.F., Krimm I., Engel M. (2019). Discovery of holoenzyme-disrupting chemicals as substrate-selective CK2 inhibitors. Sci. Rep..

[33] Laudet B., Moucadel V., Prudent R., FilholM O., Wong Y.S., Royer D., Cochet C. (2008). Identification of chemical inhibitors of protein-kinase CK2 subunit interaction. Mol. Cell Biochem..

[34] Moucadel V., Prudent R., Sautel C.F., Teillet F., Barette C., Lafanechere L., Receveur-Brechot V., Cochet C. (2011). Antitumoral activity of allosteric inhibitors of protein kinase CK2. Oncotarget.

[35] Brear P., De Fusco C., Hadje Georgiou K., Francis-Newton N.J., Stubbs C.J., Sore H.F., Venkitaraman A.R., Abell C., Spring D.R., Hyvonen M. (2016). Specific inhibition of CK2alpha from an anchor outside the active site. Chem. Sci..

[36] De Fusco C., Brear P., Iegre J., Georgiou K.H., Sore H.F., Hyvonen M., Spring D.R. (2017). A fragment-based approach leading to the discovery of a novel binding site and the selective CK2 inhibitor CAM4066. Bioorg. Med. Chem..

[37] Zhou Y., Zhang N., Qi X., Tang S., Sun G., Zhao L., Zhong R., Peng Y. (2018). Insights into the impact of linker flexibility and fragment ionization on the design of CK2 allosteric inhibitors: Comparative molecular dynamics simulation studies. Int. J. Mol. Sci..

[38] Iegre J., Brear P., De Fusco C., Yoshida M., Mitchell S.L., Rossmann M., Carro L., Sore H.F., Hyvonen M., Spring D.R. (2018). Second-generation CK2alpha inhibitors targeting the alphaD pocket. Chem. Sci..

[39] Li C., Zhang X., Zhang N., Zhou Y., Sun G., Zhao L., Zhong R. (2020). Identification and biological evaluation of CK2 allosteric fragments through structure-based virtual screening. Molecules.

[40] Bestgen B., Kufareva I., Seetoh W., Abell C., Hartmann R.W., Abagyan R., Le Borgne M., Filhol O., Cochet C., Lomberget T. (2019). 2-Aminothiazole derivatives as selective allosteric modulators of the protein kinase CK2. 2. structure-based optimization and investigation of effects specific to the allosteric mode of action. J. Med. Chem..

